# A step towards equitable clinical trial recruitment: a protocol for the development and preliminary testing of an online prostate cancer health information and clinical trial matching tool

**DOI:** 10.1186/s40814-019-0516-4

**Published:** 2019-11-07

**Authors:** Hala T. Borno, Brian M. Bakke, Celia Kaplan, Anke Hebig-Prophet, Jessica Chao, Yoon-Ji Kim, Jan Yeager, Pelin Cinar, Eric Small, Christy Boscardin, Ralph Gonzales

**Affiliations:** 1 Department of Medicine, Division of Hematology/Oncology, University of California at San Francisco, 550 16th Street, 6th Floor, Box 3211, Office 6554, San Francisco, CA 94158 USA; 2 School of Medicine, University of California at San Francisco, San Francisco, USA; 3 Department of Medicine, Division of General Internal Medicine, University of California at San Francisco, San Francisco, USA; 4 Clinical Innovation Center, University of California at San Francisco, San Francisco, USA

**Keywords:** Recruitment science, Prostate cancer, Clinical trials, Cancer disparities, Digital health

## Abstract

**Background:**

Recruitment of a diverse participant pool to cancer clinical trials is an essential component of clinical research as it improves the generalizability of findings. Investigating and piloting novel recruitment strategies that take advantage of ubiquitous digital technologies has become an important component of facilitating broad recruitment and addressing inequities in clinical trial participation. Equitable and inclusive recruitment improves generalizability of clinical trial outcomes, benefiting patients, clinicians, and the research community. The increasing prevalence of online connectivity in the USA and use of the Internet as a resource for medical information provides an opportunity for digital recruitment strategies in cancer clinical trials. This study aims to measure the acceptability, preliminary estimates of efficacy, and feasibility of the Trial Library intervention, an Internet-based cancer clinical trial matching tool. This study will also examine the extent to which the Trial Library website, designed to address the linguistic and literacy needs of broader patient populations, influences patient-initiated conversations with physicians about clinical trial participation.

**Methods:**

This is a study protocol for a non-randomized, single-arm pilot study. This is a mixed methods study design that utilizes the statistical analysis of quantitative survey data and the qualitative analysis of interview data to assess the participant experience with the Trial Library intervention. This study will examine (1) acceptability as a measure of participant satisfaction with this intervention, (2) preliminary measure of efficacy as a measure of proportion of participants with documented clinical trial discussion in the electronic medical record, and (3) feasibility of the intervention as a measure of duration of clinical visit.

**Discussion:**

The principles that informed the design of the Trial Library intervention aim to be generalizable to clinical trials across many disease contexts. From the ground up, this intervention is built to be inclusive of the linguistic, literacy, and technological needs of underrepresented patient populations. This study will collect essential preliminary data prior to a multi-site randomized clinical trial of the Trial Library intervention.

**Trial registration:**

This study has received institutional approval from the Committee of Human Subjects Research at the University of California, San Francisco.

## Background

Recruitment of a diverse participant pool to cancer clinical trials is an essential component of clinical research. Improved generalizability of clinical trials benefits patients, clinicians, and the research community [1]. For patients, participation in clinical trials may render immediate and long-term benefits. Direct participation in a clinical trial leads to favorable patient experiences with the health care system [2–4]. Moreover, participants in clinical trials tend to report higher adherence to medical care plans and trust in their physicians [5]. On a broader level, diverse participant pools in cancer clinical trials are essential to ensure meaningful clinical outcomes and to avoid missing potentially viable therapeutic interventions [6–9]. Effective therapies can be overlooked, or missed entirely, in unexamined minority populations [10].

Improved representation in clinical trials by race/ethnicity, age, and sex remains a critical need [11–13]. The vast majority of publicly funded cancer phase I–III clinical trials enroll ethnically White patients younger than 65 years old [14]. Black and Hispanic patients account for less than 5% of clinical trial participants, despite comprising 12% and 16% of the US population, respectively [11, 14]. Older adults account for approximately one third of the participants in prostate, lung, breast, and colorectal cancer trials, yet account for more than one third of the patients for these major cancer types [15, 16]. The underrepresentation of women in cancer clinical trials has been found to be especially pronounced among women over 64 years of age, who are significantly less likely to enroll in clinical trials compared to men [11, 14].

In prostate cancer, clinical trial disparities based on race/ethnicity are pronounced [17, 18]. Black men are known to have an increased risk of developing and dying from prostate cancer compared to White men [19]. Despite this alarming trend, Black men are grossly underrepresented in therapeutic clinical trials [14].

Complex socioeconomic and communication barriers are major drivers of these disparities, further compounding the difficulty that already exists in clinical trial recruitment [20, 21]. More than 30% of clinical trial sites fail to recruit even a single participant, 50% of clinical trials fail to reach their enrollment goal, and fewer than 20% of clinical trials are completed on time [22, 23]. Whereas the majority of cancer patients receive their longitudinal care in community settings, the majority of clinical trials and recruitment efforts are conducted at academic medical centers and research institutions [24], imposing logistical limitations on the patient populations that can more easily be recruited.

Most recruitment efforts for cancer clinical trials utilize a variety of recruitment strategies simultaneously, including direct recruitment by physician investigator, telephone call by research coordinator, and distributed information such as print materials and websites [25, 26]. Direct recruitment through a clinician or researcher relies on identifying potentially eligible patients from their medical records or via patient databases, as well as an understanding of clinical trial availability and eligibility criteria, a process which can be resource intensive [27]. Web-based materials compose the largest proportion of distributed recruitment material (62%) and are significantly more likely to be written at higher grade level than all other printed materials [25]. The current strategies for clinical trial recruitment leave much room for improvement.

The increasing prevalence of online connectivity via home computers and mobile phones in the USA, and use of the Internet as a resource for medical information, provides an enormous opportunity for digital recruitment strategies into cancer clinical trials [23, 28]. Recent data regarding the prevalence of Internet access in the USA revealed that 81% of all US adults have a smartphone, while 73% of US adults have access to broadband Internet in their home [29]. Among medically underserved populations, such as African Americans, Hispanics, low-income adults, and those with high school education or less, the prevalence of smartphone access is above 70% for each group [29]. Among all US adults, 46% report that smartphones were their primary means of accessing the Internet [29].

Digital interventions have the potential to overcome geographical barriers, automate patient eligibility, and deliver information to a diverse patient population [30]. The vast majority of adults in the USA use the Internet as the first source of health information, even before consulting a health care provider [31]. The use of online cancer resources is also common among minorities, and Internet use among Black and Hispanic populations has increased steadily over the last 10 years [28, 32, 33]. Among older adults, we see an increasing reliance on the Internet as a resource for medical information [31, 34, 35]. Consistent with these observations, the viability of Internet-based tools as an intervention to improve clinical trial participation among more diverse patient populations has already been supported by recent studies [34, 36, 37]. Social media and targeted advertising campaigns have also become a powerful vehicle for engaging with a diverse population [36, 37].

This pilot study aims to measure the acceptability, feasibility, and preliminary estimates of efficacy of the Trial Library intervention. The preliminary efficacy of the Trial Library will be assessed by the ability of this intervention to promote discussion of clinical trials during medical oncology clinic visits and, subsequently, its ability to encourage patient enrollment in clinical trials. Trial Library is a multicomponent web-based intervention designed as an Internet resource to provide relevant clinical trial results on broadly accessible platforms, thereby providing patients with actionable information about additional medical therapies and trials that they can discuss with their physician. The Trial Library tool has been designed to be easily accessible on any computer with Internet connectivity, and a concerted effort was made during the design process to also make Trial Library easy to navigate and user-friendly on smartphones. In this way, the Trial Library intervention seeks to improve the patient navigation experience for clinical trials. This pilot study will be performed at a single site to collect essential preliminary data prior to performing a larger multi-site randomized clinical trial of the intervention among a diverse population of men with advanced prostate cancer.

## Methods/study design

### Study design

This is a non-randomized single-arm pilot study among patients with prostate cancer seen at the Helen Diller Family Comprehensive Cancer Center (HDFCCC) at the University of California, San Francisco.

### Eligibility criteria

New patients with prostate cancer presenting to HDFCCC with a life expectancy of at least 6 months, ability to read and speak English, and fill out online forms will be considered for this study. Patients already enrolled on a therapeutic clinical trial will not be eligible for this study.

### Study schema

**Fig. 1:**
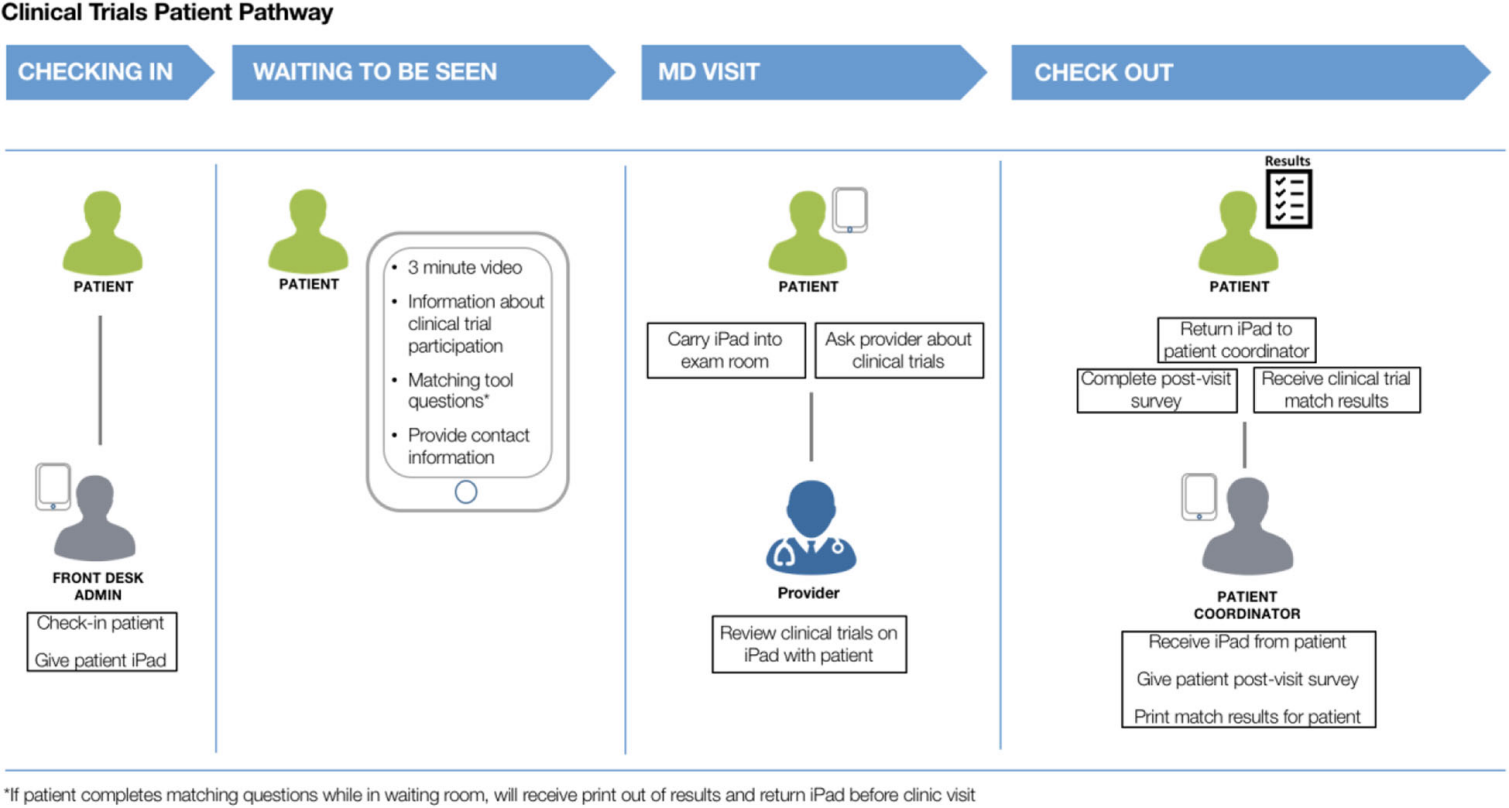
Patient clinic flow on study

The study schema is shown in Fig. 1. Potential participants meeting the study eligibility criteria will be identified by the study coordinator through electronic health record review 7 days prior to the clinic appointment. After check-in, all potentially eligible participants will be approached by a study coordinator, introduced to the research study, and asked to provide written informed consent on paper. The Institutional Review Board requires that all study participants be informed of the details of the study including any potential risks or benefits and that informed consent be acquired from each participant before they can be included in the study. This study requires a Health Insurance Portability and Accountability Act (HIPAA) form in order for researchers to access electronic health records and collect participant clinical and sociodemographic information. Informed consent for the survey is required for all participants; however, participants will also have the option to provide informed consent to participate in a qualitative interview. Required and optional consent for patient participation in components of this study are detailed in Table 1. Subsequent to providing informed consent, the participant will be given an iPad. Approached participants may decline to participate in the study and return the iPad to the check-in desk or the study coordinator. Physicians will be approached by a study coordinator to provide written informed consent on paper for a qualitative interview within 7 days of encounter with the study participant.

**Table 1:** Required and optional consent for participation in components of Trial Library study

Components of consent	Function	Requirement for participation/inclusion
Trial Library study participation	Informs patients of the purpose and content of Trial Library, risks, and benefits of participation. Allows patients to utilize Trial Library on iPad in clinic during visit.	Yes
HIPAA form	Provides permission for researchers to access patient electronic medical record for collection of participant clinical and sociodemographic information.	Yes
Qualitative interview	Informs patient of purpose of optional follow-up qualitative interview regarding patient experience using Trial Library and potential impact of this tool on their clinic visit and their understanding and/or interest in clinical trial participation.	No

### Study intervention

The Trial Library intervention has several components summarized using TiDIER format (Additional file 1) [38]. The first component is visual, audio, and written educational content describing clinical trials. The second component is a matching tool composed of a series of visual questions, such as an image of a human body with separate selections for non-metastatic versus metastatic disease. The last component is a patient report that includes all available trial options, including the technical and plain description of clinical trial name, summary of trial description in plain English, frequency of appointments required for clinical trial, and location of trial. Participants who review the components of the Trial Library intervention will receive a printed copy of a trial matching report and return the iPad to the front desk. At the end of the clinic visit, participants will be asked to complete a post-visit survey regarding their experiences using the Trial Library intervention and interactions with their physicians. A subset of participants will be identified by convenience sampling, and all clinic physicians will be asked to participate in semi-structured interviews about their experiences with the Trial Library intervention by phone or in-person. Table 2 summarizes the interview guide questions, and their associated study themes, that will be used to conduct qualitative interviews with selected participants of this study.

**Table 2:** Semi-structured interview guide summary and associated study themes

Question	Feasibility	Acceptability	Efficacy
Patient			
Tell me about your experience using the Trial Library website.	X	X	
What did you learn from using the Trial Library website?		X	X
How did using the Trial Library website influence your visit with your health care provider?	X		X
How did using the Trial Library website influence your interest in participating in clinical trials?			X
Physicians			
Tell me about your experience having the Trial Library pilot study take place in your clinic.	X	X	
What is the clinical value of the Trial Library resource?		X	X
How did using the Trial Library website influence your visit with your patients?	X		X

### Intervention development

Initial development of the Trial Library intervention and subsequent development of this study protocol are the result of a collaborative effort of researchers, product and service design specialists, engineers, and consumers. Input from patients with prostate cancer during the design process, via patient interviews, has been instrumental in understanding how to create a tool that is accessible and informative. The primary end consumers of the Trial Library intervention are patients and caregivers. The goal of the development of this protocol is to propose methodology for developing and piloting technological interventions for equitable recruitment into clinical trials based on patient-centered design models while also considering clinical, sociological, and technological perspectives.

Information about clinical trials provided in this resource is intended to be useful, relevant, and accessible to all prostate cancer patients, while also aiming to address the informational needs of vulnerable and underrepresented patients. These aims were addressed in the product development process applying human-centered design methods and tools. Human-centered design incorporates the human perspective across a structured process to solve problems. In developing the Trial Library intervention, researchers first conducted a focus group of Black men with prostate cancer to gather information about what was considered to be relevant and desirable in a resource, and what beliefs and knowledge gaps existed about clinical trials that should be addressed in the design. Focus group findings were translated into the product features. User testing was then performed in clinic waiting rooms. A total of 35 men with prostate cancer, 66% of whom self-identified as Black, provided input in the development of the Trial Library intervention. Interviewees were shown select portions of Trial Library intervention and were asked a series of open-ended questions designed to capture patient understanding of clinical trials and to assess the clarity of the clinical trial matching questions. The feedback gathered from these interviews was used to refine the product in preparation for this study. In this study, the human perspective was gathered through interviews conducted with Black men with prostate cancer, and the information gathered from those interviews informed the specific design goals and content parameters of the Trial Library intervention. Table 3 illustrates themes identified from the focus group analysis.

**Table 3:** User testing design principles

Design principles	Outputs
Design for flexibility and accessibility	Provide all website features at surface level (educational articles, matching tool, misc. resources) Universal navigation menu Contact info to Nurse Navigator for additional help
Be transparent and honest	Content is written in clear, concise plain language Provide disclaimer(s) re: accuracy of presented info
Having a “trustworthy” voice is better than a “legitimate” one	Content covers information that fits contextually to patients’ lifestyle and beliefs User testimonies
Facilitate a “safe space”	Conversational, empathetic tone Provide clear directions on how to access matching tool, but not in a forceful manner

### Methods

This is a mixed methods study design and will include quantitative surveys and qualitative interviews to assess the participant and physician experience with the Trial Library intervention. This study will also measure preliminary estimates of efficacy of the Trial Library intervention in promoting patient-physician discussion of clinical trials. Efficacy will be determined by whether a clinical trial discussion was documented in the electronic medical record during the clinic visit. All procedures have been approved by the UCSF Institutional Review Board for research on human subjects.

### Measures and statistical analysis

Patients will complete a self-administered survey on paper at the end of the clinic visit with multiple-choice questions (Additional file 1). The survey will include brief demographic questions about the patient’s education level and health literacy. Demographic characteristics will be collected from electronic medical record, including date of birth, race/ethnicity, preferred language, and zip code. Additionally, demographic characteristics of participants in the study will be compared to the overall demographic characteristics of patients with prostate cancer seen at HDFCCC to assess representativeness of the sample. Demographic and clinical characteristics will be summarized by descriptive statistics.

### Quantitative survey

The primary objective of this study is acceptability which will be a measure of overall satisfaction rate. Patients will respond to a set of multiple-choice questions on paper at the end of the clinic visit (Additional file 1). The survey will include a question about participant satisfaction with the study intervention and how the use of this intervention impacted their visit with their provider. In order to minimize human transcription error when inputting data from paper to a computer database, the study will use double entry verification.

### Sample size and justification

The sample size for this study is 66 participants. In the literature, 35% of patients with cancer utilize the Internet as a preferred source of health information over their provider [39]. To our knowledge, there is no data available on satisfaction rate with online health information; therefore, we use 35% as a proxy for satisfaction rate with online health information in this patient population. To measure the overall satisfaction rate of study participants, we will determine the proportion of participants who report some degree of satisfaction on a 5-item Likert scale from extremely dissatisfied to extremely satisfied with the Trial Library intervention by point estimation and its 95% confidence interval and will be compared to a proxy reference satisfaction rate of 35% by one-sample proportion test. In this pilot feasibility study, the null hypothesis that the true satisfaction rate (acceptability) is 35% will be tested against a one-sided alternative. The null hypothesis will be rejected if 29 or more satisfactions are observed in 66 participants. This design yields a type I error rate of 0.0487 and power of 0.8010 when the true satisfaction rate is 50%.

### Electronic medical record review

A secondary objective of this study is to obtain a preliminary estimate of efficacy. In this study, preliminary efficacy will be based on the extent to which the Trial Library intervention promotes discussion of and recruitment to clinical trials. The endpoints of clinical trial discussion documentation will be measured by electronic medical record review. Recruitment to clinical trials will be measured through electronic medical record review approximately 30 days after the participant is enrolled on the Trial Library intervention study.

In the literature, 34% of patients receiving usual care without additional online health information about cancer clinical trials had discussion about clinical trials based on audio recordings of the oncologist consultation [40]. Given that the literature lacks a baseline rate of documentation of clinical trial discussion, 34% will be used as the reference in this study. To evaluate if the Trial Library intervention increases the proportion of participants with documented clinical trial discussion, a one-sample proportion test will be used compared to a reference of 34%.

### Qualitative semi-structured interview

An exploratory objective of this study is feasibility. Feasibility will be measured through semi-structured interviews and clinical data, such as impact on duration of visit and wait time. As described in Table 2, semi-structured interviews will be performed with a subset of participants (10–12) and all practice physicians seeing eligible patients (6) to assess feasibility and acceptability of the intervention. Interview participants will be identified through convenience sampling. Semi-structured interviews involve use of open-ended questions and probes to elicit descriptive data. Most studies suggest saturation, or retrieval of no new information, is achieved with 8 participants [41]. In this study, telephone interviews will be conducted approximately 7 days after the participant enrolls in the Trial Library study. Physician interviews will occur in-person after clinic visits or within 7 days of encounter with participant. The interviews will use open-ended questions, and participant responses will be recorded, transcribed, and analyzed using thematic analysis. An analysis of saturation will be performed with two researchers coding transcripts of interviews. The researchers will meet to compare and reconcile code, discuss discrepancies, and achieve consensus on each transcript. If saturation is not achieved, up to 8 additional participants may be recruited for interview.

Moreover, data will be extracted from the real-time locating system for hospitals (RTLS), a location technology platform currently instituted as part of standard of care at HDFCCC. RTLS is a system used to provide immediate or real-time tracking and management of staff and patients. This data will be utilized to evaluate the impact that in-clinic use of the Trial Library tool has on the duration of each clinic visit. These measures will be used to assess the feasibility of this intervention. While in the clinic, all patients and doctors wear a locating sensor which is used to gather data on location and visit duration. Investigators will collect participant wait time and length with provider during clinic visit from the RTLS data. The data gathered for participants will be compared to a historical baseline generated by 66 patients who meet the study eligibility criteria but who presented to the clinic prior to the initiation of this study.

To evaluate if the Trial Library intervention increases clinic length time, a two-sample *t* test will be used to compare minute average of study participants with the reference group (deidentified minute average of new patients seen at HDFCCC prior to initiation of study).

### Data management

All data for this study will be entered by a researcher using Research Electronic Data Capture (REDCap) software (Vanderbilt University). REDCap data forms incorporate range checks for data values. REDCap offers a data Export Utility which will facilitate regular data quality checks. This database will improve data integrity by minimizing risk of data loss, providing a secure environment, and reducing transcription error. All audio recordings will be stored in a secure, password-protected computer. Audio recordings will be transcribed verbatim without participant identifiers. Transcripts and interview notes will be reviewed by the study team for general themes.

## Discussion

This novel study leverages health information technology to develop an online informational resource for a diverse patient population. The principles that informed the design of the Trial Library intervention aim to be generalizable to clinical trials across many disease contexts. From the ground up, this resource is built to be inclusive of the linguistic, literacy, and technological needs of underrepresented patient populations.

This study will measure the acceptability of the Trial Library intervention among patients with prostate cancer seen at one academic medical center. The qualitative component of this mixed methods study will capture the participant and physician experience with the intervention. This study will provide critical preliminary data prior to a larger multi-site randomized clinical trial of the intervention. Positive results from this study would support the use of Internet-based recruitment tools as a viable method for recruiting diverse patient populations into clinical trials within a clinical setting. Additionally, preliminary data from this study may inform larger interventions delivering online health information to improve clinical trial recruitment in the community.

This study will also examine the physician perspective after incorporating a patient online trial resource in the clinical context. Given that this intervention is being tested in a closed system, the clinic, we will be able to assess the impact of the intervention on the patient and physician clinical experience. It is possible implementation of this study intervention may disrupt workflow and increase clinic length. This study is targeting new patients and measuring clinic visit time to capture these effects.

We anticipate limitations in this single site pilot study and will only capture preliminary estimates of efficacy. The intervention will only populate one clinical site’s available trials and be available in English. Future studies will include a translated copy of the website in Spanish and incorporate trial options for multiple clinical sites.

The Trial Library intervention is intended to build on current recruitment methods and to be a resource that is informative, accessible, and user-friendly for all prostate cancer patients. Trial Library is designed to be an Internet-based cancer clinical trial matching tool that enables patients to view information about prostate cancer clinical trials for which they may be eligible based on individual characteristics of their disease process. This tool provides patients with general information about clinical trials, as well as information specific to each trial for which the patient may be eligible. Designing a user interface and navigation platform that embraces the touch screens of mobile phones and tablets was an important part of our efforts to reach broader patient populations. In order to achieve this, we approached the development of the Trial Library intervention simultaneously from clinical, sociological, and technological perspectives. The design process involved gathering and integrating valuable input from clinicians and clinical trial recruitment literature, patients from traditionally marginalized and underserved groups, and human-centered design specialists. Results from this pilot study will inform future studies to evaluate the efficacy of a patient-centered online matching tool in achieving equitable recruitment to prostate cancer clinical trials.

## Supplementary information

**Additional file 1.** Intervention Description Checklist using TiDIER Format and Trial Library Post Visit Survey

## Data Availability

Not applicable

## Abbreviations

HDFCCC: Helen Diller Family Comprehensive Cancer Center
RTLS: Real-time locating system
UCSF: University of California, San Francisco
