# Supplementary material for: A step towards equitable clinical trial recruitment: a protocol for the development and preliminary testing of an online prostate cancer health information and clinical trial matching tool
Source: Pilot Feasibility Stud. 2019 Nov 7;5:123. doi: 10.1186/s40814-019-0516-4 (PMC6839161; doi:10.1186/s40814-019-0516-4)
Supplement: Supplementary file 1 — Additional file 1. Intervention Description Checklist using TiDIER Format and Trial Library Post Visit Survey [file 40814_2019_516_MOESM1_ESM.docx]

**Appendix**

1. Intervention Description Checklist using TiDIER Format

| The Trial Library Intervention | |
| --- | --- |
| 1 | The Trial Library Intervention |
|  | Patient-facing, multicomponent web-based prostate cancer clinical trial matching tool |
| Purpose of Intervention | |
| 2 | Improve the patient navigation experience for patients identifying clinical trials by assisting potential clinical trial participants in using the internet to identify clinical trials |
| Intervention Components | |
| 3 | iPad will be provided to the participant with the web-based intervention loaded. The web-based intervention will include visual, audio, and written educational content describing clinical trials. |
| 4 | Procedures included in the intervention include: (1) a survey and matching tool composed of a series of visual questions, such as an image of a human body with separate selections for non-metastatic versus metastatic disease, (2) video describing clinical trials and research, and (3) available trial matching report. |
| 5 | Matching report will be printed for the patient that includes all available trial options, including the technical and plain description of clinical trial name, summary of trial description in plain English, frequency of appointments required for clinical trial, and location of trial. |
| Intervention Modality | |
| 6 | Mode of delivery of the intervention is via the internet |
| Intervention Context | |
| 7 | The intervention will occur at HDFCCC |
| 8 | The intervention will be delivered daily from Mon-Fri 8am-5pm at HDFCCC until study fully accrues |
| Intervention Tailoring and Adapting | |
| 9 | This intervention is not intended to be adapted during the study period |
| 10 | This intervention is not intended to be modified during the study period |
| 11 | RTLS data and a post-visit survey will be used to assess intervention adherence or fidelity |

**2. Trial Library Post Visit Survey**

1. Who was the main provider you saw in the clinic today?

Nurse

Nurse Practitioner

Fellow with Attending Physician

Attending Physician

2. Did you discuss clinical trials with your health care provider today?

Yes

No🡪 please skip to Question 4

I do not remember

3. If yes, who initiated the discussion about clinical trials?

Your Provider

Your Nurse

You (Patient)

Friend or Family Member

Other

4. Did you match to any trials on the *Trial Library*?

Yes

No

I do not remember

5. Overall, how satisfied or dissatisfied are you with *Trial Library*?

Extremely dissatisfied

Dissatisfied

Neither satisfied nor dissatisfied

Satisfied

Extremely satisfied

6. How likely is it that you would recommend *Trial Library* to a friend or family member?

Extremely likely

Somewhat likely

Neither likely nor unlikely

Somewhat unlikely

Extremely unlikely

7. Did your experience with the *Trial Library* increase your interest in participating in a clinical trial?

Yes

No

8. What is the highest degree you have earned?

High School or GED

Vocational

Associate's Degree

Bachelor's Degree

Master's Degree

Doctorate

9. How often do you need someone to help you when you read instructions, pamphlets, or other written materials from your doctor or pharmacy?

Never

Rarely

Sometimes

Often

Always

10. Are you willing to be contacted by phone within the next 7 days for a brief follow-up interview about your experience today with the *Trial Library* website?

Yes

No

11. What time of the day are you most likely to be available for a follow-up telephone interview?

Morning

Afternoon

Evening
